# OsGATA16*,* a GATA Transcription Factor, Confers Cold Tolerance by Repressing *OsWRKY45–1* at the Seedling Stage in Rice

**DOI:** 10.1186/s12284-021-00485-w

**Published:** 2021-05-12

**Authors:** Hongjia Zhang, Tao Wu, Zhao Li, Kai Huang, Na-Eun Kim, Ziming Ma, Soon-Wook Kwon, Wenzhu Jiang, Xinglin Du

**Affiliations:** 1 Jilin Province Engineering Laboratory of Plant Genetic Improvement, College of Plant Science, Jilin University, No. 5333 Xi’an Road, Changchun, 130062 China; 2 Department of Plant Bioscience, College of Natural Resources and Life Science, Pusan National University, Milyang, 50463 Republic of Korea

**Keywords:** Rice, Transcription factor, OsGATA16, Haplotype, Cold tolerance

## Abstract

**Background:**

Cold stress is the main abiotic stress in rice, which seriously affects the growth and yield of rice. Identification of cold tolerance genes is of great significance for rice to solve these problems. GATA-family transcription factors involve diverse biological functions, however, their role in cold tolerance in rice remains unclear.

**Results:**

In this study, a GATA-type zinc finger transcription factor *OsGATA16*, which can improve cold tolerance, was isolated and characterized from rice. OsGATA16 belongs to OsGATA subfamily-II and contains 11 putative phosphorylation sites, a nuclear localization signal (NLS), and other several conserved domains. *OsGATA16* was expressed in all plant tissues, with the strongest in panicles. It was induced by cold and ABA treatments, but was repressed by drought, cytokinin and JA, and acted as a transcriptional suppressor in the nucleus. Overexpression of *OsGATA16* improves cold tolerance of rice at seedling stage. Under cold stress treatments, the transcription of four cold-related genes *OsWRKY45–1*, *OsSRFP1*, *OsCYL4*, and *OsMYB30* was repressed in *OsGATA16-*overexpressing (OE) rice compared with wild-type (WT). Interestingly, OsGATA16 bound to the promoter of *OsWRKY45–1* and repressed its expression*.* In addition, haplotype analysis showed that *OsGATA16* polarized between the two major rice subspecies *japonica* and *indica*, and had a non-synonymous SNP8 (336^G^) associated with cold tolerance.

**Conclusion:**

OsGATA16 is a GATA transcription factor, which improves cold tolerance at seedling stage in rice. It acts as a positive regulator of cold tolerance by repressing some cold-related genes such as *OsWRKY45–1*, *OsSRFP1*, *OsCYL4* and *OsMYB30*. Additionally, OsGATA16 has a non-synonymous SNP8 (336^G^) associated with cold tolerance on CDS region. This study provides a theoretical basis for elucidating the mechanism of cold tolerance in rice and new germplasm resources for rice breeding.

**Supplementary Information:**

The online version contains supplementary material available at 10.1186/s12284-021-00485-w.

## Introduction

Rice (*Oryza sativa* L.) is an important staple food crop that provides sustenance for more than half of the global population (Fairhurst and Dobermann 2002; Tang et al. 2019). Rice production is confined to certain cultivation regions due to its temperature sensitivity, and rice crops experience frequent environmental stresses, such as extremes of temperature, drought, and high salinity, which risk declines in the quality and abundance of rice production (Hussain et al. 2018; Kumar et al. 2014). The increase of global populations and the consequent increase in food demand have prompted the expansion of rice production to less-suitable cultivation areas, and increased the probability of rice crops being subjected to severe environmental stresses (Zhang et al. 2017). For example, low temperatures in China reduced rice yield by 3–5 hundred million tons, with severe impacts on grain security (Zhang et al. 2017; Zhu et al. 2015). The optimal temperature for rice growth is 26–30 °C, and the impacts of exposure to cold temperatures vary with the growth stages: at the seedling stage, it affected physiological metabolism (Zhang et al. 2014); at the booting stage, it adversely affected the fructification percentage (Jiang et al. 2010); and exposure at the flowering and pollination stages, it affected the percentages of the pollination and fructification (Shinada et al. 2013; Shakiba et al. 2017). The genetic and molecular basis of cold tolerance in rice is therefore an area of active research due to its practical relevance.

Plants respond to stresses, such as cold exposure, by activation of internal stress defense mechanisms that stimulate physiological responses. For example, overexpression of several stress-responsive genes, including *OsAPX1* and *OsiSAP8*, resulted in physiological changes that improved cold tolerance (Sato et al. 2011; Kanneganti and Gupta 2008). The rice cold signaling pathway is an area of active research, and one study identified COLD1 as a novel rice cold sensor. A SNP at this locus conferred cold tolerance in *japonica* rice, and COLD1 was found to interact with a G-protein subunit and expedite GTPase activity (Ma et al. 2015). The plant hormone ABA was also found to be involved in the cold signaling pathway (Vishwakarma et al. 2017; Ma et al. 2009; Park et al. 2009; Fujii et al. 2009; Kim et al. 2012). Under cold stress, ABA levels were found to increase and stimulate binding of the ABA receptor PYR to PP2C, thus repressing PP2C binding to SnRK2. SnRK2 then phosphorylated other TFs, activating the expression of ABA responsive genes and increasing cold stress tolerance. Additionally, other diverse TFs involved in cold tolerance have been identified including bZIPs (Liu et al. 2012; Shimizu et al. 2005; Zou et al. 2008; Hossain et al. 2010), WRKYs (Kim et al., 2016a; Yokotani et al. 2013), ZFPs (Liu et al. 2007; Huang et al. 2009; Zhang et al. 2012), and TCPs (Yang et al. 2013; Wang et al., 2014), which had positive or negative effects on cold tolerance in rice.

In plants, *trans*-acting factors interacted with specific *cis*-acting elements in the promoters of the target genes according to their diverse functional activities to activate or repress gene expression (FrancoZorrilla et al., 2014). As a member of TF family, OsGATA protein, contains highly conserved structure in rice genome and is responsible for regulating a series of plant functions (Gupta et al. 2017). It contains a GATA-type zinc finger domain (C-X2-C-X (17–20)-C-X2-C) located near the DNA-binding domain (Gupta et al. 2017), and can bind to the specific sequence of XGATAY (X is T or A, Y is G or A) in the promoter region of the target gene (Reyes et al. 2004). Reyes et al. (2004) identified about 30 GATA genes from rice and *Arabidopsis thaliana*, and divided them into four classes according to the numbers and locations of the introns and exons, including A, B, C, and D. In addition, Gupta et al. (2017) identified 28 OsGATAs, and divided them into seven subfamilies (subfamilies-I, II, III, IV, V, VI, VII) according to their gene structure and the number and positions of GATA domains. In addition, according to the structural features, subfamily-II can be divided into Class-A and Class-B: three Class-A proteins (*OsGATA9*, *OsGATA14*, and *OsGATA15*) contain a highly conserved HAN domain at the N-terminal; six Class-B proteins (*OsGATA8*, *OsGATA10*, *OsGATA11*, *OsGATA12*, *OsGATA13*, and *OsGATA16*) contained an LLM domain at the C-terminal**.** Although the functions of GATA-family proteins have been explored in fungi and animals for a long time, they have not been discovered in plants until recently (Tsai et al. 1994; Scazzocchio 2000; Tong et al. 2000; Zhang and He 2018). Their functions were related to flowering, metabolism, leaf growth, organelle development and responses to plant hormones (Richter et al. 2010; Richter et al. 2013; Chiang et al. 2012; Hudson et al. 2013; Zhang et al. 2015; He et al. 2018).

Although many studies have shown the involvement of GATA family transcription factor in abiotic stress responses, their precise biological function and molecular mechanisms in rice remain to be elucidated. In this study, we reported the identification of *OsGATA16*, a GATA family transcription factor in rice. We found that *OsGATA16* expression was induced by cold stress, and its overexpression in transgenic rice improved cold tolerance at seedling stage. Our results showed that *OsGATA16* might have an important role in cold tolerance of rice.

## Results

### OsGATA16 Encodes a GATA Class-B TF

**Fig. 1:**
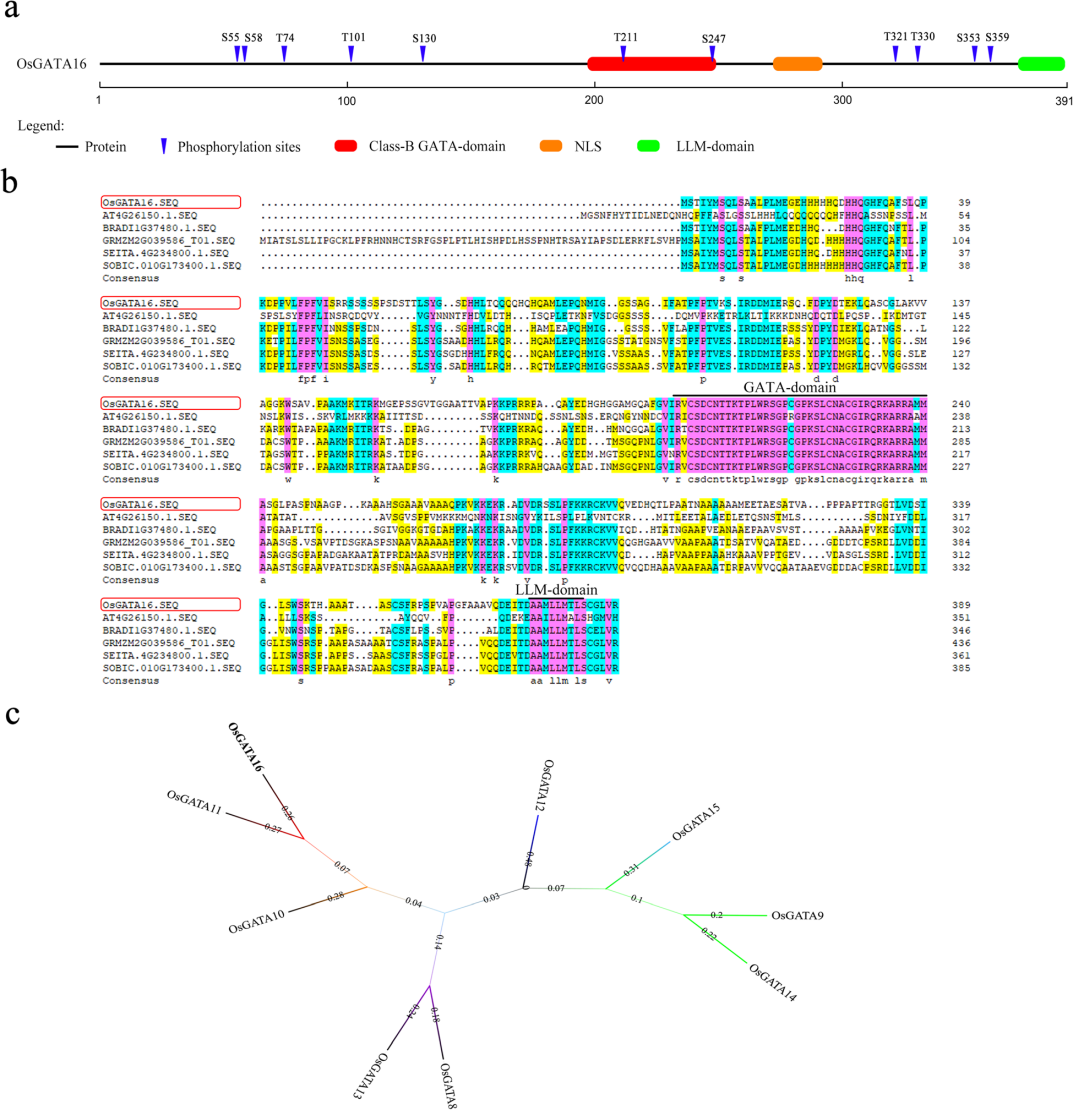
Bioinformatic analysis of OsGATA16 protein. **a** Structure of OsGATA16 protein. Numbers correspond to locations within the full-length protein. **b** Comparison of *OsGATA16* homologous genes in various species. Black lines represent diverse domain, pink represents identical amino acid residues, and similar amino acid residues are highlighted in blue and yellow. **c** Phylogenetic tree of OsGATA subfamily-II proteins. Numbers represent level of approximation

A GATA-type zinc finger Transcription factor, OsGATA16 (LOC_Os06g37450; NCBI name: OsGATA22), which related to cold stress was identified through bioinformatic screening. OsGATA16 belongs to Class-B of GATA subfamily-II (Behringer and Schwechheimer 2015), and contains 11 cold-related putative phosphorylation sites, a highly conserved GATA-type zinc finger domain, an LLM domain as well as a NLS (Fig. 1a). It contains 391 amino acids with a pI of 9.82 and MW of 41.1 KDa. Comparison of *OsGATA16* homologous genes in diverse plant species (*Brachypodium distachyon*, *Setaria italic*, *Sorghum bicolor*, *Zea mays*, and *Arabidopsis thaliana*) revealed that the GATA zinc finger domains were highly conserved over substantial evolutionary time (Fig. 1b). Phylogenetic analysis of subfamily-II genes using MEGA7.0 showed that *OsGATA16* was most similar to *OsGATA11* (Fig. 1c).

### Transcriptional Analysis of *OsGATA16*

The *OsGATA16* promoter region (2000 bp upstream of the initial ATG) was analyzed to gain insights into the biological function of OsGATA16. Using online tools (https://sogo.dna.affrc.go.jp), several putative *cis*-regulatory elements related to abiotic stress and hormones were found, including cold-responsive elements, dehydration responsive elements, ABA responsive elements, salt induced elements, and cytokinin responsive elements (Table 1). We speculated that *OsGATA16* might be associated with abiotic stress and hormone responses via transcriptional regulation mechanisms in rice.

**Table 1:** Putative cis-acting elements in the *OsGATA16* promoter

Name	Sequence	Position	Annotation
LTRECOREATCOR15	CCGAC	17,153,960	cold response
EBOXBNNAPA	CANNTG	30,139,689,696,712,1386	cold response
ABRERATCAL	MACGYGB	688,734,1795	ABA response
ABRELATERD1	ACGTG	1731,1796,1821	dehydration response
MYBCORE	CNGTTR	889,972,1035	dehydration response
CPBCSPOR	TATTAG	1453,1667	cytokinin response
ARR1AT	NGATT	80,90,120,257,266,557,592,633,843,848,861,920,929,935,982,997,1012,1044,1062,1071,1077,1087,1135,1180,1200,1236,1361,1396,1628,1682	cytokinin response
GT1GMSCAM4	GAAAAA	1250,1298,1655,1765	salt-induce

**Fig. 2:**
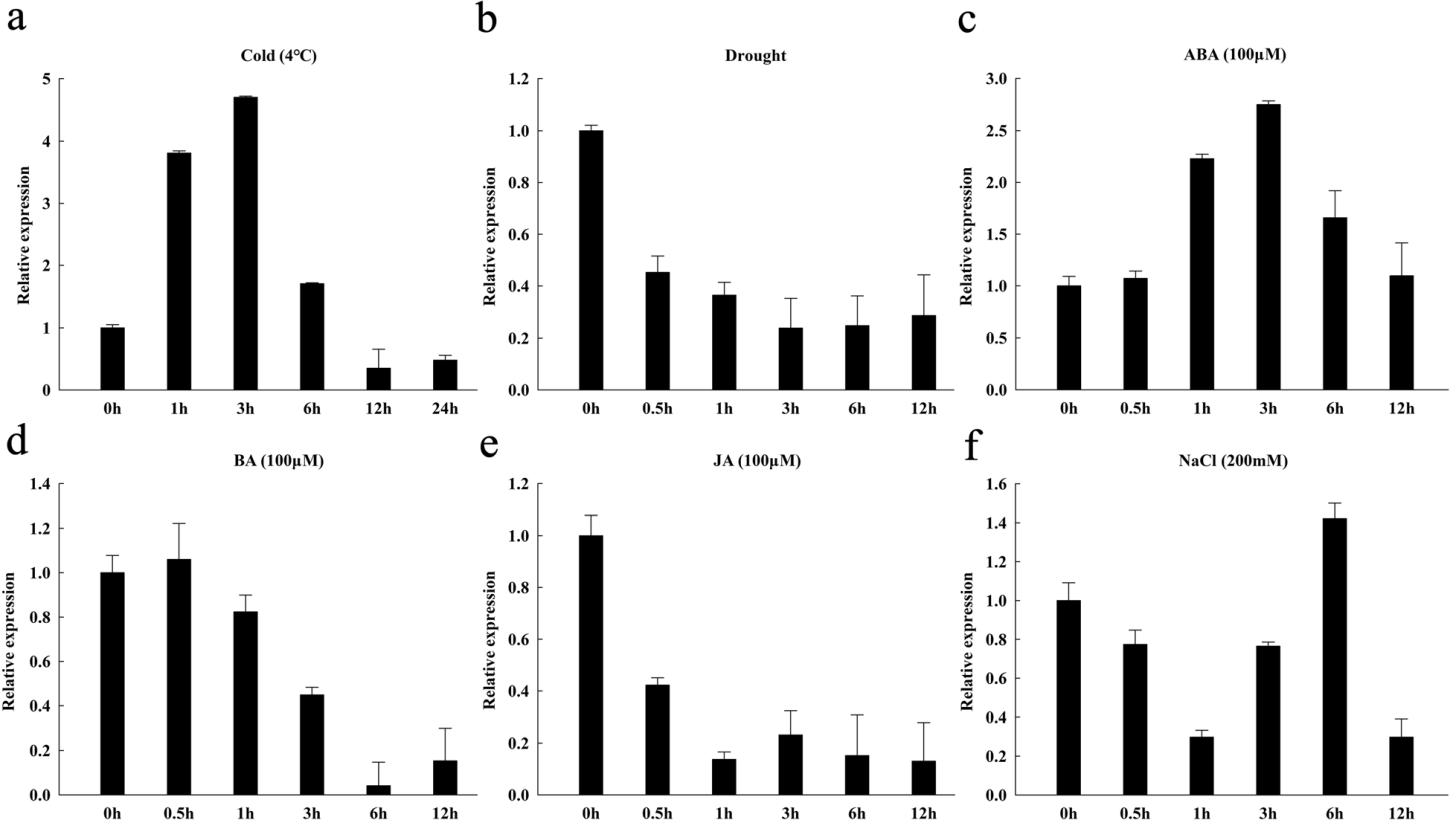
Time-course expression analysis of *OsGATA16* after exposure to abiotic stress or phytohormones. **a** Cold, **b** Drought, **c** Abscisic acid (ABA), **d** 6-benzylaminopurine (BA), **e** Jasmonic acid (JA), and **f** NaCl. Data represent the mean ± SE from three replicates

To investigate the possible involvement of *OsGATA16* in stress responses, transcriptional expression of *OsGATA16* was examined under abiotic stress (cold, drought, high salinity) conditions and plant hormone (ABA, 6-BA, and JA) treatments. *OsGATA16* expression was induced by cold and ABA treatments, but suppressed by drought, 6-BA, and JA treatments (Fig. 2). *OsGATA16* expression increased within 3 h of exposure and then decreased gradually under cold and ABA treatments (Fig. 2a and c), however it was rapidly and substantially repressed within 0.5 h followed by maintenance of low expression levels under drought and JA treatments (Fig. 2b and e). *OsGATA16* expression was repressed more slowly within 6 h under BA treatment, (Fig. 2d). Interestingly, it was more complex after exposure to high concentration of NaCl, with an increase following initial repression (Fig. 2f).

**Fig. 3:**
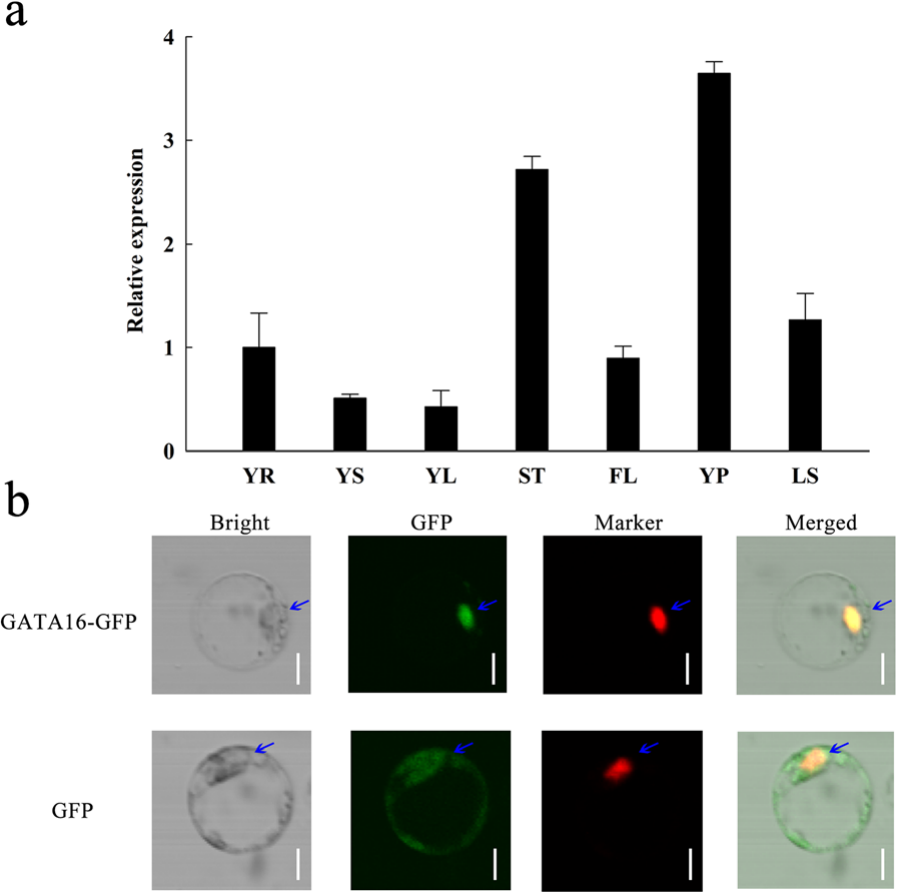
Tissue-specific expression and subcellular localization of OsGATA16 in rice. **a** *OsGATA16* expression in different plant tissues. Root (YR), stem (YS), and leaves (YL) at the seedling stage, and stem (ST), flag leaves (FL), panicles (YP), and leaf sheaths (LS). Data represent the mean ± SE from three replicates. **b** Subcellular localization of OsGATA16 in rice. GATA16-GFP: GFP fusion with OsGATA16 protein; D53-RFP: nuclear marker. Arrows indicate nuclei. Bar = 10 μm

In addition, temporal and spatial expression of *OsGATA16* was also tested in diverse plant tissues from different rice growth stages. *OsGATA16* was expressed in all plant tissues, including young roots, stems, leaves at the seedling stage, stems, flag leaves, panicles and leaf sheaths at the booting stage. Expression was the most abundant in the panicles, followed by stems, leaf sheaths, young roots and flag leaves (Fig. 3a).

### *OsGATA16* Overexpression Improves Cold Tolerance in Rice

To elucidate whether the biological function of *OsGATA16* confers cold tolerance, OE plants were generated. The full coding region of *OsGATA16* under the control of the maize ubiquitin promoter was introduced into the *japonica* rice variety of Kitaake via *Agrobacterium*-mediated transformation (Fig. S1a), and two independent OE lines (OE-1 and OE-2) were obtained (Fig. S1c). qRT-PCR analysis showed that transcriptional expression of *OsGATA16* in OE-1 and OE-2 lines was up-regulated substantially compared with WT plants under normal growth conditions (Fig. S1b).

**Fig. 4:**
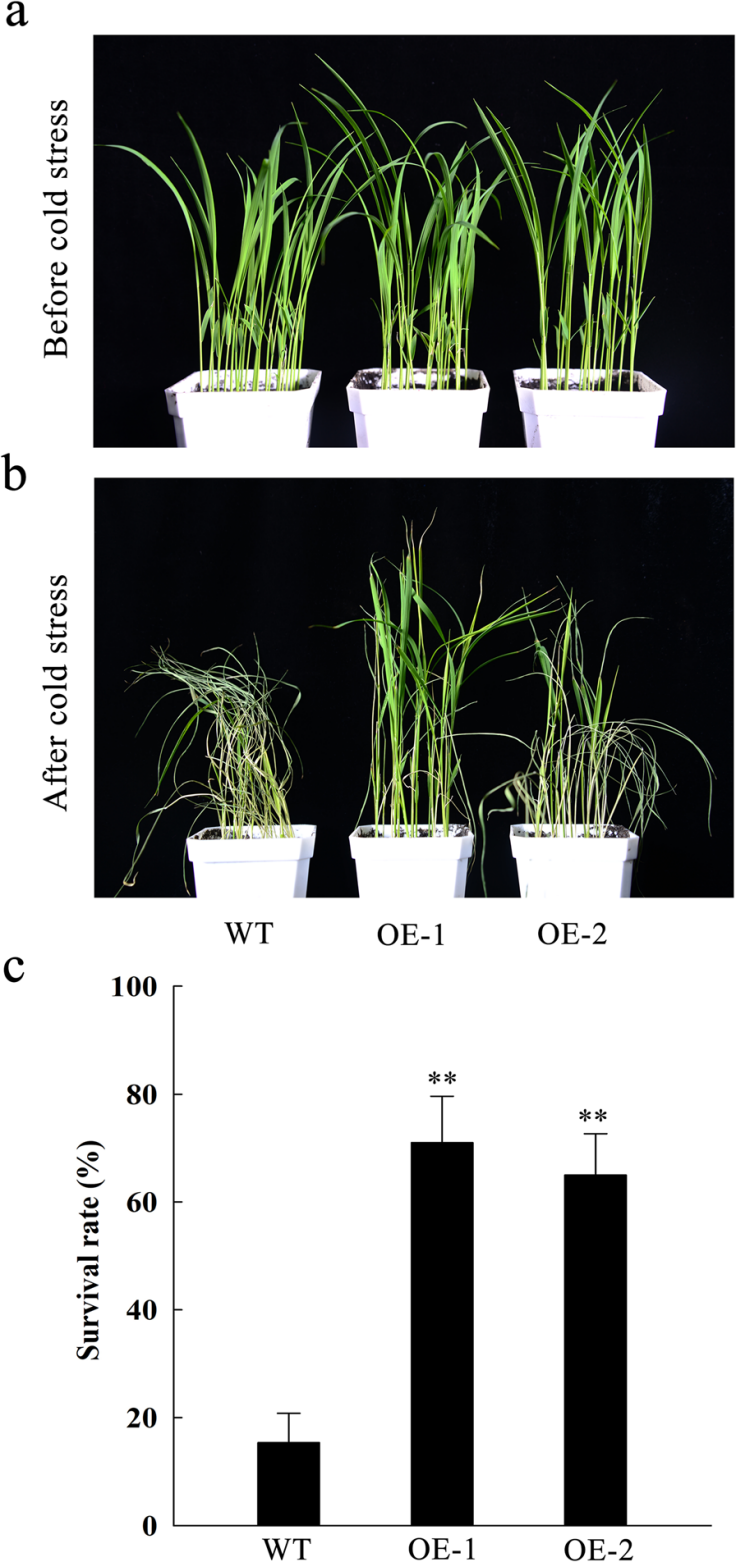
*OsGATA16* overexpression phenotype and survival rate after exposure to cold stress. **a** Wild-type (WT) and *OsGATA16-*overexpression (OE) lines at the 3-leaf seedling stage, prior to exposure to cold stress (8 °C). **b** WT and OE seedlings after cold stress exposure and recovery at room temperature. **c** Survival of OE and WT plants after exposure to cold stress. Data represent the mean ± SE from five replicates. Asterisks indicate significant differences in survival rate (Student’s t-test, **p < 0.01)

Cold stress treatments were performed at the seedling stage in OE and WT. Rice plants were grown at a low temperature (8 °C) for 7 days and then allowed to recover at a normal temperature (28 °C) for 7 days. OE and WT seedlings displayed no apparent phenotypic differences before cold treatment (Fig. 4a), but clear differences were observed after treatment (Fig. 4b). OE-1 and OE-2 plants showed significantly higher survival rates compared with WT plants (Fig. 4c). These results suggested that overexpression of *OsGATA16* in rice could improve cold tolerance at seedling stage.

Additionally, rice agronomic traits were evaluated under normal field cultivation conditions, and no obvious differences for plant height and hundred-grain weight were observed between OE and WT plants (Fig. S2).

### Nuclear Localization and Transcriptional Activity of OsGATA16

The OsGATA16 protein contains a basic NLS region (KVKKEKRADVDRSSLPFKKRC), which can be predicted to function in the nucleus. To determine the subcellular localization of OsGATA16, an OsGATA16-GFP fusion was constructed under the control of the ubiquitin promoter and was co-transformed into rice protoplast cells alongside a nuclear localization marker, D53-RFP (Zhou et al. 2013). As shown in Fig. 3b, GFP signal was observed in both the cytosol and nucleus of GFP-independent group, whereas the OsGATA16-GFP fusion protein localized predominantly at the nucleus. This indicates that OsGATA16 primarily functions within the rice cell nucleus.

**Fig. 5.**
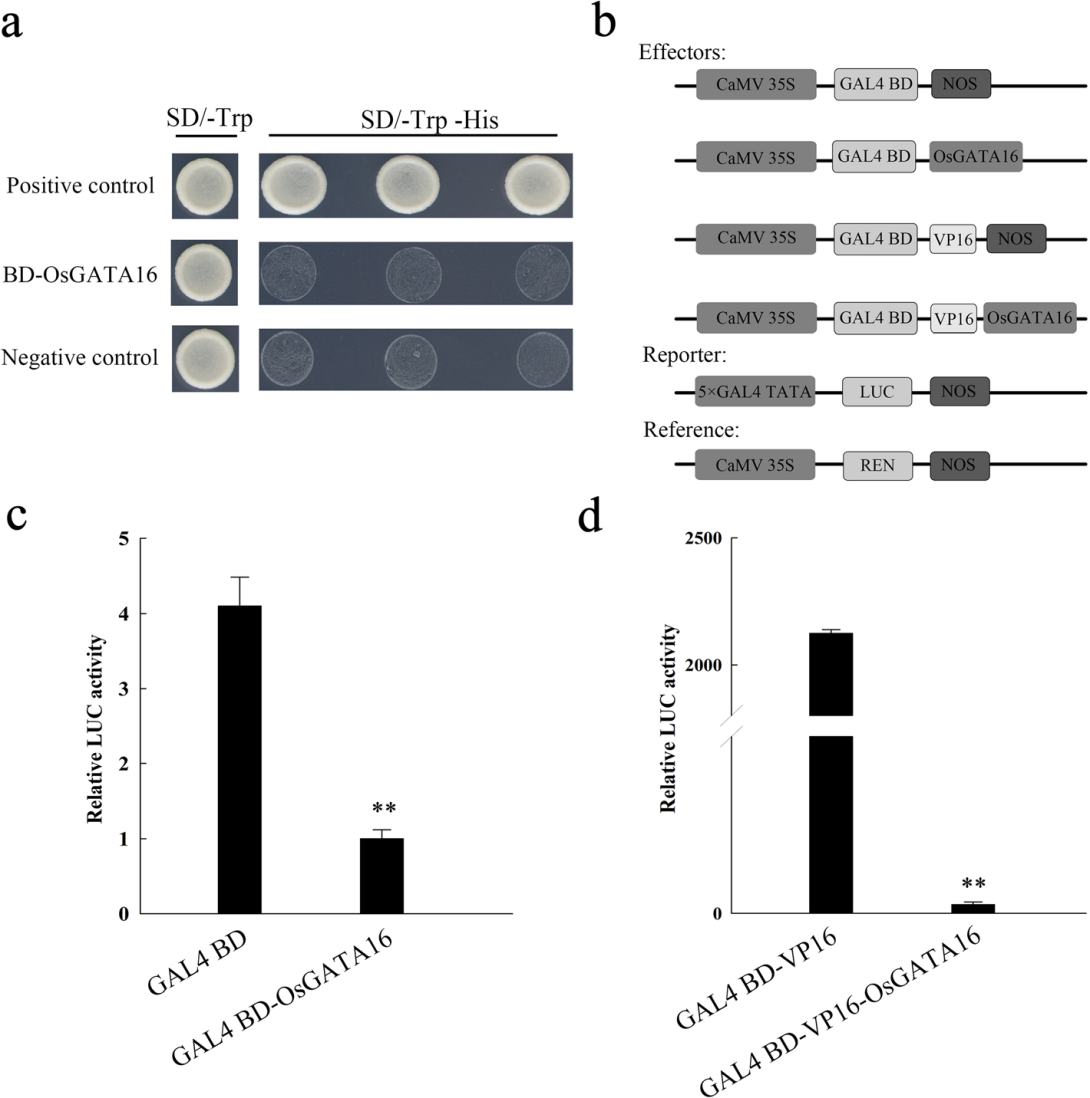
Transcriptional activity of OsGATA16 in rice. **a** Yeast two-hybrid analysis of OsGATA16 transcriptional activity. **b** Schematic representation of recombinant effector, reporter, and reference (pPTRL) plasmids for Dual-luciferase reporter analysis. **c-d** Relative LUC activity with control and OsGATA16 effector constructs. Data represent the mean ± SE from three replicates. Asterisks indicate significant differences in relative LUC activity (Student’s t-test, **p < 0.01).

Yeast two-hybrid and Dual-luciferase reporter assays were used to assess the transcriptional activity of OsGATA16. In two-hybrid analysis, the positive control exhibited growth on SD/−Trp and SD/−Trp-His media (Fig. 5a). The experimental target (BD-OsGATA16) results were consistent with the negative control (BD), with normal growth on SD/−Trp medium and inhibited growth on SD/−Trp-His medium, indicating transcriptional repression (Fig. 5a). In the dual-luciferase assay, effector and reporter plasmids were co-transformed into rice protoplast cells, with pPTRL (REN luciferase) used as an internal reference (Fig. 5b). As shown in Fig. 5c and d, LUC luciferase activities were significantly repressed in the experimental target (GAL4 BD-OsGATA16) compared with the control (GAL4 BD). Furthermore, when OsGATA16 was fused with the VP16 activation domain, the LUC luciferase activity of the target group (GAL4 BD-VP16-OsGATA16) was repressed > 2000-fold compared with the control (GAL4 BD-VP16). Taken together, these results indicate that OsGATA16 acted as a transcriptional suppressor, and this activity was intensified by the VP16 transactivation domain.

### Overexpression of OsGATA16 Prompts Down-Regulation of Cold-Related Genes

Cold-sensitive genes were identified in the NCBI database, and a subset of genes was used to explore the cold-related signal transduction mechanism of OsGATA16 in rice. Expression of cold-related genes was differed between OE and WT plants under normal and cold stress treatments conditions, as determined using qRT-PCR with specific primers (Table 2). Four cold-sensitive genes, *OsWRKY45–1*, *OsSRFP1*, *OsCYL4*, and *OsMYB30*, were down-regulated in OE lines under both conditions (Fig. S3). These results suggest that OsGATA16 acted as a cold-related functional factor and was involved in cold signaling pathways by direct or indirect regulation of these candidate genes.

**Table 2:** Primers for qRT-PCR

Gene ID	Name	Forward primer	Reverse primer
LOC_Os12g44350	*Actin*	CCTGGCAGTATGAAGGTAGTTG	GAAGCACTTCATGTGGACGAT
LOC_Os06g37450	*OsGATA16*	TGCTTGAGCCCCAAAATATG	GCAGCTTCTCGGTATCGTAT
LOC_Os01g10840	*OsGSK1*	ACGGGTCACATCATCTCC	AGTTCCTACAACTCGCTCC
LOC_Os03g08570	*OsPDS*	ACTGGCTGCCTGTCATCT	TACTTGCGAAGCACCTAT
LOC_Os05g25770	*OsWRKY45–1*	GCAGCAATCGTCCGGGAATT	GCCTTTGGGTGCTTGGAGTTT
LOC_Os05g49890	*OsRAN2*	TGGTGGACTTAGGGATGG	GGAATGTGACCTGCTTGG
LOC_Os02g10920	*OsSRZ1*	ATGAACAGGAAGCCAGGAGACT	TCCACCGAAGGAGGAACCA
LOC_Os01g55940	*OsGH3–2*	GAAGATGAGCTGGACAGGAGGC	GGGCGGTGCTTGAAGTGAT
LOC_Os06g45140	*OsbZIP52*	GCGAATAAGAAGGATGGTGTC	GCTTGAAGAGGGATGAGTTTT
LOC_Os03g22680	*OsSRFP1*	ATTCGGCAGGATGGGATT	TCGTGGACTCGTTGTGGC
LOC_Os09g02270	*OsCYL4*	GACCTCGCCATCCTCAAC	AACTCGCCGAACTCCTTT
LOC_Os02g10200	*OsZFP185*	CCAAGTGCCACAAGGAGAT	CCCACCGTCACAACCATT
LOC_Os02g41510	*OsMYB30*	ACAACACCACGGACAGTTTCAC	CCGTCATTGCCAGCGTCT
LOC_Os07g05720	*OsTCP21*	CACGCGGAGATGACGCACTA	ACCCACAAGACCCGAGGACA
LOC_Os01g11550	*OsPCF5*	TCCAGAGCTACACGCCTGACC	ATGGCGATGTTGCTGGTGG
LOC_Os03g57190	*OsTCP8*	CATGTCCTCGGGTTTCTTGGG	GCTGCTGCTGATGGTGGTGG
LOC_Os10g28600	*OsTCD10*	GCCTGGTTTATTTCCTTG	GTCTCGACATCCCTCCTC
LOC_Os12g42190	*OsPCF8*	CCGTCGCTCGACTGCTCCTT	GGCTTGCTGCCGTTGGTGTT

### OsGATA16 Suppresses the *OsWRKY45–1* Promoter

GATA proteins are thought to bind to *cis*-regulatory elements containing GATA motifs (XGATAY). Several XGATAY elements were found in the promoter regions of four cold-responsive genes whose expression was repressed by *OsGATA16* overexpression (Table 3). This suggests that OsGATA16 might bind to the promoters of these candidate genes and regulate their transcription as part of the cold signaling pathway.

**Table 3:** Putative XGATAY motifs in promoters of candidate genes

Name	Sequence	Position
*OsWRKY45–1pro*	XGATAY	134,517,660
*OsSRFP1 pro*	XGATAY	562,567,755,976,1006,1350
*OsCYL4 pro*	XGATAY	210
*OsMYB30 pro*	XGATAY	1241,1468

**Fig. 6:**
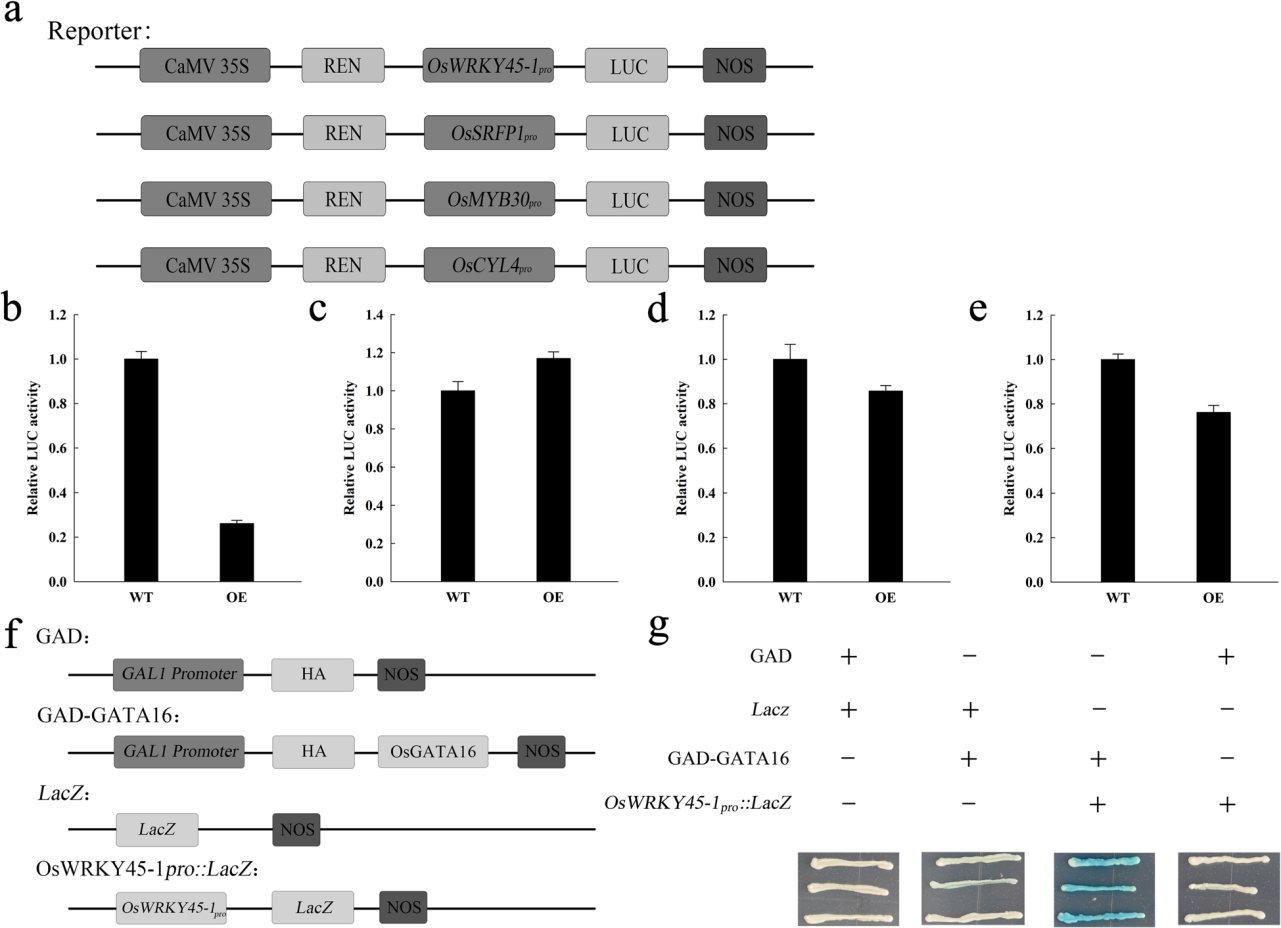
OsGATA16 interaction with the *OsWRKY45–1* promoter in rice. **a** Schematic representation of reporter plasmids for Dual-luciferase reporter analysis, with REN luciferase as an internal control **(b-e)** Relative LUC activity in wild-type and *OsGATA16*-overexpression (OE) lines with **(b)** *OsWRKY45–1*, **(c)** *OsSRFP1*, **(d)** *OsCYL4*, and **(e)** *OsMYB30* reporter constructs*.* Data represent the mean ± SE from three replicates. **(f)** Schematic representation of recombinant plasmids for yeast one-hybrid analysis of the *OsWRKY45–1* promoter. **(g)** Yeast one-hybrid assay results. Blue coloration is indicative of protein–promoter interaction

Yeast one-hybrid and Dual-luciferase reporter assays were used to assess OsGATA16 binding to the promoters of the candidate genes. Several reporter constructs were generated for the rice in vivo Dual-luciferase assay, with the LUC and REN luciferase genes under the control of candidate and *35S* promoters, respectively (Fig. 6a). Under the control of the *OsWRKY45–1* promoter, LUC luciferase activity was substantially repressed in OE lines compared with WT (Fig. 6b). By contrast, the *OsSRFP1* promoter exhibited slight activation (Fig. 6c), and the *OsMYB30* and *OsCYL4* promoters showed slight repression in the OE lines compared with WT (Fig. 6d and e). These results suggest that OsGATA16 represses the *OsWRKY45–1* promoter in rice. To further examine this interaction, effector and reporter constructs were generated for yeast one-hybrid analysis of the *OsWRKY45–1* promoter (Fig. 6f). Positive interactions in the experiment were indicated by a chromogenic reaction due to LacZ expression on growth medium containing X-Gal (SD/−Trp/−Ura/+X-Gal). As shown in Fig. 6g, independent expression of GAD or LacZ (negative controls) resulted in no blue coloration, whereas the OsGATA16-*OsWRKY45–1*_*pro*_ combination resulted in a strong chromogenic response, confirming their interaction (Fig. 6g). Taken together, OsGATA16 protein directly binds to the promoter of *OsWRKY45–1* and represses its expression.

### Haplotype and Functional SNP Analysis of OsGATA16

**Fig. 7:**
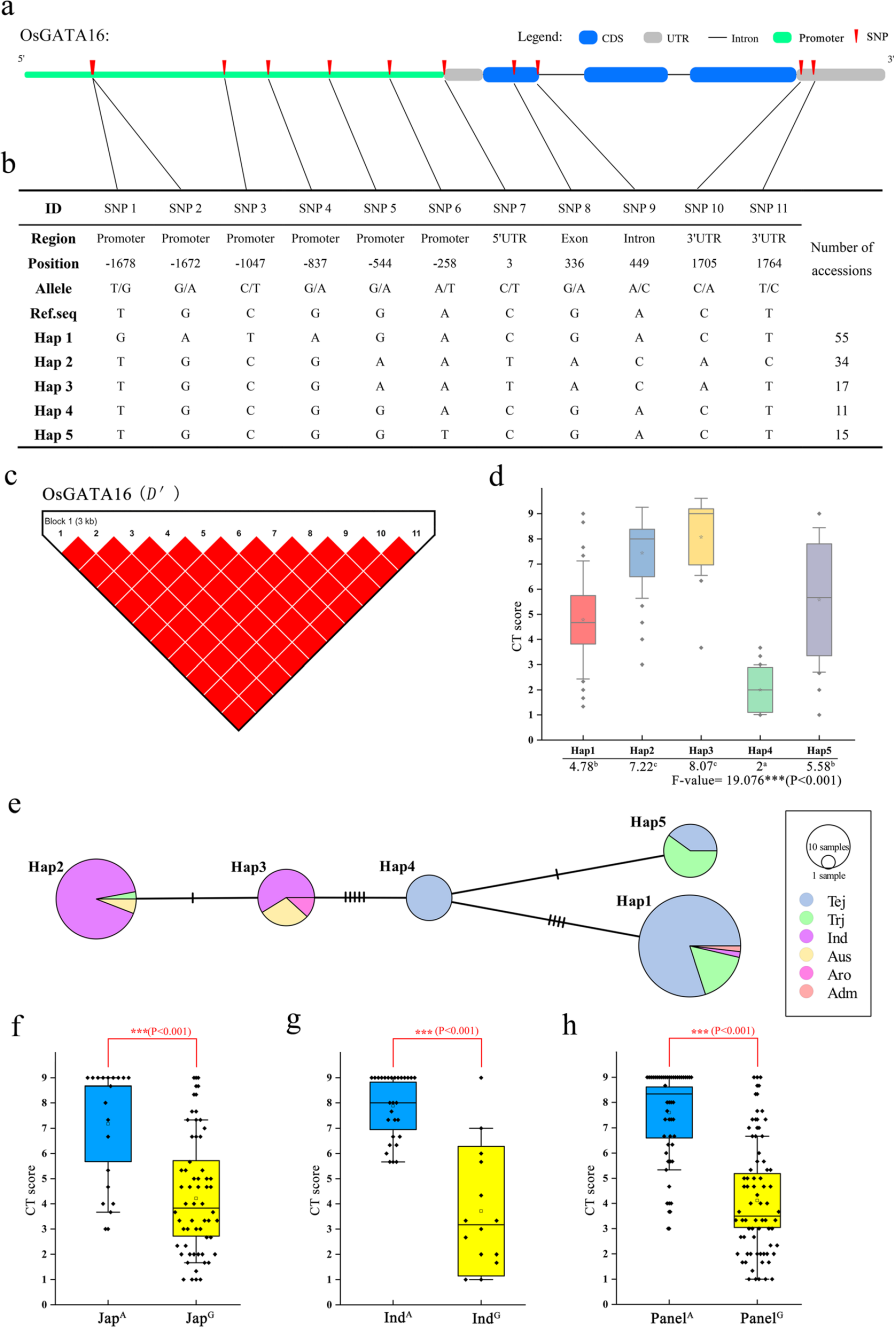
Haplotype analysis of *OsGATA16.* **a** Structural representation of *OsGATA16* and upstream promoter region*.* **b** *OsGATA16* SNPs and haplotype groups in 137 rice accessions*.* SNP positions are given relative to the start of the 5′UTR. Hap: haplotype **(c)** Linkage disequilibrium (LD) analysis of *OsGATA16*. The eleven SNPs shown in **(b)** were used for LD block assessments, with SNP numbers as in **(b).** *D′* was used for evaluation of LD. Red indicates complete linkage equilibrium between each SNP. **d** Relationship of cold-tolerant phenotype with haplotype. Cold-tolerance (CT) score is on a 1–9 scale, with 1 representing highest CT. Different letters indicate significant CT differences among haplotypes (ANOVA, Duncan test) **(e)** Haplotype network variation. Circle size represents the number of accessions in each Hap, and the number of transverse lines between each Hap represents the number of nucleotide variations. Tej, *temperate japonica*; Trj, *tropical japonica*; Ind, *indica*; Aus, *aus*; Aro, *aromatic*; and Adm, *admixture* rice varieties. **f-h** SNP 8 haplotype relationship with CT. Superscript A and G indicate the A and G genotypes in *japonica* **(f)**, *indica* **(g)**, and *japonica* plus *indica* **(h)** accessions. Asterisks indicate significant differences in CT between genotypes (Student’s t-test, ***p < 0.001)

Re-sequencing data encompassing approximately two million high-quality SNPs from a collection of 137 rice accessions was used for haplotype analysis (Kim et al., 2016b; Zhang et al. 2020). SNP positions and the structure of the *OsGATA16* gene are shown in Fig. 7a, with haplotype (Hap) information shown in Fig. 7b. In total, 11 SNPs were found in the promoter, UTR, intron, and exon regions of *OsGATA16* in five Haps. Linkage disequilibrium (LD) showed that 11 SNPs in the 3 kb gene region showed strong LD relationships between the SNP pairs (Fig. 7c). Phenotype–haplotype relationships were analyzed, with a CT score (1–9 scale) used as the evaluation index. As shown in Fig. 7d, Hap2 and Hap3 exhibited significantly higher scores than the other Haps, indicating that these two Haps were associated with higher sensitivities to cold. Hap1 and Hap5 had CT scores that were lower than Hap2 and Hap3 but higher than Hap4, with Hap4 showing the highest tolerance for cold stress (Fig. 7d). The haplotype network and variation relationships of each Hap were assessed. Consistent with the phenotypic analysis, the first group comprised Hap1, Hap4, and Hap5 and contained most of the *japonica* varieties, and the second group comprised Hap2 and Hap3 and contained most of the *indica*, *aus*, and *aromatic* varieties (Fig. 7e). Hap2 and Hap3 were closely related, as were Hap4 with Hap5, with only single SNP differences. However, Hap3 differed from Hap4 by five SNPs, and Hap4 also exhibited a distant relationship with Hap1 (Fig. 7e). These results indicate that the *OsGATA16* gene is polarized between *japonica* and *indica*.

Of the eleven identified SNPs, SNP 8, is a non-synonymous SNP (AGT to AAT) in exon, and this results in an amino acid change from serine to aspartic acid. The SNP 8 haplotype was thus assessed for its association with cold tolerance. The SNP8 336^A^ and 336^G^ genotypes were found in both *japonica* and *indica* varieties, and the 336^G^ genotype was associated with higher cold tolerance in both subspecies. When considered together, *japonica* and *indica* cultivars with the 336^A^ genotype had an average CT score of 7.6, and those with the 336^G^ genotype had an average score of 4.11 (Fig. 7h). Independent consideration of the *japonica* and *indica* varieties yielded similar results, with the 336^G^ genotype having average CT scores of 4.21 and 3.71, and the 336^A^ genotype having average scores of 7.18 and 7.89, for *japonica* and *indica* varieties, respectively (Fig. 7f and g).

The haplotype grouping of eleven SNPs in *OsGATA16* suggested a possibility of different biological functions in *japonica* and *indica* varieties. However, SNP 8 was associated with cold tolerance in both rice subspecies, and the 336^G^ genotype may enhance the function of OsGATA16 during the cold response, thus conferring enhanced tolerance.

## Discussion

TFs play important roles in diverse stress signaling pathways through regulating stress responsive genes in plants. Recent researches have explored the various functions of GATA TFs in rice, but their impact on cold tolerance has not been explored. Bioinformatic and transcriptional expression analysis of rice OsGATA family proteins demonstrated their involvement with abiotic stress responses, and several genes showed duplicated relationships and similar expression patterns during rice growth (Gupta et al. 2017). Similarly, GATA proteins in the Chickpea were shown to be involved in the response to ABA and Drought stress (Niu et al. 2020). These recent studies of GATA TFs suggest that members of the GATA gene family may be generally involved in responses to abiotic stress. In this study, a transcription factor, OsGATA16, was identified and characterized which improves cold tolerance in rice.

Several *cis*-acting elements were found in the promoter of *OsGATA16* (Table 1), as well as a range of TF binding sites such as WRKY, MYB, ABRE, and bHLH. A transcriptional study of a MYB TF, OsMYB4, involved in responses to stress, highlighted a regulatory network that facilitated the cold stress signaling pathway through mediate MYB, bZIP, NAC, ARF, ERF, and CCAAT-HAP TFs, and its overexpression also impacted panicle development (Park et al. 2010). OsRAN1, an evolutionarily conserved member of the small G-protein family, was found to have a significant role in improving cold tolerance in rice. It was reported that both of *OsGATA16* and *OsRAN1* expressed ubiquitously in rice tissues and exhibited the highest expression in panicles (Xu and Cai 2014). These results are consistent with our findings that *OsGATA16* overexpression conferred improvement of cold tolerance, and the highest *OsGATA16* expression was found in panicles. Transcriptional expression of *OsGATA16* was induced by cold and ABA treatments, suppressed by drought, BA, and JA treatments (Fig. 2), and was especially expressed the strongest in in panicles (Fig. 3a). OsGATA16 may associate with other TFs in panicle tissues to mediate responses to cold exposure as well as to other abiotic stresses and phytohormones.

OsGATA16 was localized to the nucleus and acted as a transcription repressor. Transcription of cold-sensitive genes, *OsWRKY45–1*, *OsSRFP1*, *OsCYL4*, and *OsMYB30*, was repressed in OsGATA16-OE lines compared with WT (Fig. S3), and OsGATA16 suppressed the promoter activity of *OsWRKY45–1*(Fig. 6). Previous studies have reported that OsWRKY45–1 was involved in response to low temperature, ABA, salt and drought stress (Tao et al. 2011). Combining our results of *OsGATA16* induction under cold, salt and ABA treatment (Fig. 2) with previous studies (Tao et al. 2011), we hypothesized that *OsGATA16* may improve cold tolerance by repressing the expression of the cold-sensitive gene *OsWRKY45–1* through ABA signaling pathway. In addition, recent research has identified several new features of OsWRKY45. The *OsWRKY45–1* and *OsWRKY45–2* alleles encode proteins that differed by 10 amino acids, and are related to rice diseases (Tao et al. 2009). The two alleles exhibited opposite roles in resistance to bacterial blight caused by *Xoo* and bacterial leaf streak caused by *Xoc*. Overexpression of *OsWRKY45–1* reduced resistance to *Xoo* and *Xoc*, but increased resistance to rice blast caused by the fungus *Magnaporthe grisea*. The response to *Xoo* infection was accompanied by increased accumulation of SA and JA (Tao et al. 2009). Interestingly, the expression of *OsGATA16* was repressed by JA exposure (Fig. 2), and also repressed by *Xoo* infection which was demonstrated by microarray analysis (Kong et al. 2020). From these results, we can infer that OsGATA16 may be related to the disease resistance by repressing *OsWRKY45–1* expression: When the disease is infected, the increase of JA level can repress the expression of *OsGATA16*, leading to the repression of *OsWRKY45–1*, thus improve the disease resistance. Further analysis is needed to determine whether *OsGATA16* confers disease resistance as well as its mechanisms.

GATA-family TF proteins are highly conservative. Most members of the family maintain a GATA-type zinc finger protein domain proximal to the DNA-binding domain (Behringer and Schwechheimer 2015). The functions of GATA-family TFs were associated with cytokinin, nitrate, and light responses. OsGATA16 is the most evolutionarily similar to OsGATA11 (Cga11) (Fig. 1). *Cga11* is induced by cytokinin and confers chloroplast development and plant growth (Hudson et al. 2013). Interestingly, however, *OsGATA16* is repressed by cytokinin (Fig. 2). Promoter analysis showed that there were two copies of cytokinin-response motif CPBCSPOR in both *OsGATA11* and *OsGATA16*. However, the copy number of another cytokinin-response motif, ARR1AT, is different in both genes: 16 in *OsGATA11* and 31 in *OsGATA16* (data not shown). These differences may lead to the differences in the cytokinin response of two homologous genes. The real function of *OsGATA16* in cytokinin pathway needs to be clarified in the further study.

Rice subspecies, *japonica* and *indica*, exhibit polarization for many agronomic traits, including adaptation and resilience to low temperatures (Ma et al. 2015). *japonica* varieties generally display better tolerance to cold stress than *indica* varieties, due to evolutionary adaptations to growth in regions with different climates (Wang et al., 2014). Some cold-related genes may have retained their ancestral functions in older varieties, but environmental adaptations may have supported the persistence of novel alleles with different functions in cultivated rice varieties that have been further selected and preserved by breeding processes (Kim et al., 2016a). For example, *OsbZIP73*, which is involved in the ABA-dependent cold signaling pathway, harbors a single SNP between *japonica* and *indica* varieties. The SNP is located in an exon and leads to an amino acid disparity that partially explains differences in cold tolerance between subspecies (Liu et al. 2018). An SNP (SNP2) in *COLD1*, a novel cold sensor in rice, was highly variable among diverse subspecies, but was conserved in *japonica* varieties and was associated with cold tolerance in cultivated rice (Ma et al. 2015). Haplotype analysis of *OsGATA16* showed that some novel alleles were associated with different subspecies (Fig. 7): Eleven SNPs were identified within a strong LD block, five Haps were distinguished according to SNP variation, and *japonica* and *indica* varieties were clearly defined in two separate groups. Phenotypic analysis showed that the *indica* group was significantly more cold-sensitive than the *japonica* group. A non-synonymous functional SNP (SNP 8, 336^A/G^) was significantly associated with cold tolerance in both *japonica* and *indica* varieties when considered separately or together. As with *OsbZIP73* and *COLD1*, OsGATA16 showed clear differentiation between rice subspecies and conferred cold tolerance in rice. Furthermore, the 336^G^ allele was significantly associated with cold tolerance in both *japonica* and *indica* varieties, and has potential as a novel functional allele for improving cold tolerance in rice breeding programs.

## Conclusion

In the present study, we identified a GATA transcription factor OsGATA16, which plays a positive role in cold tolerance of rice seedlings. OsGATA16 represses cold-related genes (*OsWRKY45–1*, *OsSRFP1*, *OsCYL4*, and *OsMYB30*) and binds to *OsWRKY45–1* promoter. In addition, it has a non-synonymous SNP8 (336^G^) associated with cold tolerance. The role of OsGATA16-OsWRKY45 interaction and SNP8 in the mechanism of cold tolerance will be further elucidated in next study.

## Materials and Methods

### Bioinformatics Evaluation of *OsGATA16*

For bioinformatics analysis, protein sequences were obtained from the NCBI and analyzed using NCBI protein BLAST. Putative phosphorylation sites were predicted using an online tool at http://gps.biocuckoo.org (Wang et al. 2020), and promoter sequences were assessed using an online tool at https://sogo.dna.affrc.go.jp. Alignment of *OsGATA16* homologous genes from diverse species was performed using DNAMAN, and an evolutionary tree was constructed using software MEGA7.0, NJ-tree function was used with repeats of 1000-bootstraps.

### Growth Conditions, Stress Treatments, and Expression Patterns

WT seeds and T_3_ seeds of *OsGATA16* OE lines were germinated for 3 days at room temperature. Geminated seeds were sown in soil in pots and cultivated under a 16 h light/8 h dark cycle at 26 °C/28 °C with 65% humidity. To test the induction of *OsGATA16* expression by stress treatments, rice seedlings at the 3-leaf stage were treated with cold (4 °C), drought (dehydrated at 28 °C with 65% humidity), 200 mM NaCl, 100 μM ABA, 100 μM BA, or 100 μM JA. Young roots, stems, seedling-stage leaves and stems, flag leaves, young panicles, and booting stage leaf sheaths were collected for expression pattern analysis.

### RNA Isolation and Quantitative Real-Time PCR

Total RNA was isolated from stress-treated rice seedlings and diverse tissues of rice using an RNA Prep Pure Plant Kit (Tiangen, Beijing, China) according to the manufacturer’s instructions. RNA (2 μg) was reverse transcribed to cDNA using a RevertAid RT Reverse Transcription Kit (Thermo Scientific, Waltham, USA), and qRT-PCR was conducted using an Agilent Stratagene Mx3005P Quantitative Real-Time PCR system with SGExcel FastSYBR qPCR Mixture (Sangon Biotech, Shanghai, China). The *β-actin* gene was used as an internal qRT-PCR control. Sequences for gene-specific primers are shown in Table 2. Three replicate experiments were performed for each sample. The relative quantitation method (*ΔΔCT*) was used to evaluate quantitative variation among replicates.

### Subcellular Localization

The *OsGATA16* coding region lacking the stop codon was inserted into the 1305-Ubi-GFP vector between the *Kpn*I and *BamH*I restriction sites, in-frame with GFP under the control of the ubiquitin promoter. D53-RFP, which targets to the nucleus, was used as a co-localization marker (Zhou et al. 2013). The expression construct and localization marker (10 μg) were transiently co-transformed into rice protoplasts and incubated overnight in darkness at 28 °C, as described previously (Chen et al. 2006). Fluorescence was observed with a Zeiss LSM780 confocal laser microscope (Carl Zeiss, Germany).

### Construction of Pubi:*OsGATA16* Plasmid and Generation of Overexpressing Rice Lines

Full-length *OsGATA16* cDNA was amplified from total RNA from *Oryza sativa subsp. japonica* cv*.* Kitaake and inserted into the modified vector pCAMBIA1301. The resulting overexpression vector, Pubi:*OsGATA16*, was introduced into Kitaake (WT) by *Agrobacterium-*mediated transformation (Hiei et al. 1994). T_3_ homozygotic OE and WT Kitaake lines were selected for analysis.

### Evaluation of Cold Tolerance

Cold stress treatments were performed at the seedling stage in OE and WT. Rice plants were grown at a low temperature (8 °C) for 7 days and then allowed to recover at a normal temperature (28 °C) for 7 days. Survival rates were determined after 14- day treatments by counting the surviving plants (leaf contains about 40% green part) and dead plants.

### Dual-Luciferase Reporter Assay

Recombinant effectors GAL4 DB-OsGATA16 and GAL4 DB-VP16-OsGATA16 were constructed and co-transformed into rice protoplasts alongside reporter 35S-GAL4-LUC, with pPTRL (*Renilla reniformis* luciferase) as a reference control. For protein-DNA-binding analysis, the promoters of each candidate gene were inserted into the pGreenII vector, and the constructed *promoter::LUC* vectors were transformed into WT and OE lines. After incubation overnight at 28 °C in darkness, protoplasts were examined using the Dual-Luciferase® Reporter Assay System (Promega), according to the manufacturer’s instructions. Luminescence was detected using a GloMax® Discover Multimode Microplate Reader (Promega).

### Yeast One-Hybrid Assay

The full-length coding region of *OsGATA16* was inserted into the pJG4–5 vector between the *EcoR*I and *Xho*I restriction sites and named GAD-OsGATA16, with Trp1 within the vector acting as a selection marker. The *OsWRKY45–1* promoter was inserted into the pLacZi2μ vector between the *EcoR*I and *Sal*I sites and named as *Promoter*::*LacZ*, with Ura3 and *LacZ* in the vector acting as selection markers. The two recombinant plasmids were co-transformed into the EGY48 yeast strain and cultivated on SD medium lacking Trp and Ura and containing X-Gal (SD/−Trp-Ura + X-Gal) at 30 °C for 2 days. The blue color of the chromogenic reaction was indicative of protein-DNA binding.

### Yeast Two-Hybrid Assay

The OsGATA16 coding sequence was purified by PCR and cloned into the pBridge (BD) vector to yield BD-OsGATA16, which was then transformed into the Y2HGold yeast strain and cultivated on yeast medium. Empty BD vector was used as a negative control. Transformed strains were initially cultivated on SD medium without Trp (SD/−Trp) to select transformants, and then transferred to SD medium without Trp and His (SD/−Trp-His) to assess the transcriptional activity of OsGATA16.

### Haplotype Analysis

Genotype and phenotype data were collected for haplotype analysis. Genotype data were collected and filtered as described previously (Kim et al., 2016b; Zhang et al. 2020). Phenotype data comprised evaluation scores for cold tolerance during the seedling stage. Rice was cultivated under field conditions and irrigated with cold water (13 °C) up to 10 days, followed by normal temperature recovery for 1 week. Plants were scored 1–9 according to their sensitivity to cold stress, with 9 = most resistant. For haplotype analysis, SNPs within the *OsGATA16* genic and promoter regions (upstream 2000 bp) were identified, and their position relative to the start of the 5′ UTR was recorded. Haplotype grouping followed SNP variation in each haplotype (Hap). Only samples without missing data and without heterozygosis were used for haplotype analysis. Visualization of haplotype variation analysis was performed using PopArt software, LD block analysis was performed using Haploview software.

## Supplementary Information

**Additional file 1: Fig. S1.** Construction of *OsGATA16* overexpression transgenic lines. **(a)** Schematic of recombinant overexpression plasmid with *OsGATA16* under the control of a ubiquitin promoter. **(b)** Expression analysis of *OsGATA16* in wild-type (WT) and overexpression (OE) lines by qRT-PCR. Data represent the mean ± SE from three replicates. Asterisks indicate significant differences in expression level (Student’s t-test, **p < 0.01). **(c)** Verification of transgenic OE lines by PCR. NC: negative control; M: 2000 bp marker. **Fig. S2.** Analysis of agronomic traits in wild-type (WT) and *OsGATA16-*overexpression (OE) lines under field conditions. **(a)** WT and OE plants at maturity. **(b-c)** Trait statistics for plant height **(b)** and hundred-grain weight **(c)** in WT and OE lines at maturity. Data represent the mean ± SE from three replicates. **Fig. S3.** Expression analysis of cold-sensitive genes in wild-type and *OsGATA16-*overexpression (OE) lines by qRT-PCR under normal and cold conditions. Data represent the mean ± SE from three replicates.

## Data Availability

All data generated or analyzed during this study are included in this published article and its supplementary information files.

## Abbreviations

LLM: Leucine-Leucine-Methionine
HAN: Hanaba Taranu
NLS: Nuclear localization signal
OE: Overexpression transgenic line
WT: Wild type
CT: Cold tolerance
qRT-PCR: Quantitative real-time PCR
ABA: Abscisic acid
BA: Cytokinin
JA: Jasmonic acid
GFP: Green fluorescent protein
RFP: Red fluorescent protein
LUC: Firefly luciferase
REN: Renilla luciferase
TF: Transcription factor
Xoo: Xanthomonas oryzae pv. oryzae
Xoc: Xanthomonas oryzae pv. Oryzicola
*M. grisea*: Magnaporthe grisea
Trp: Tryptophane
Leu: Leucine
His: Histidine
Ura: Uracil
X-gal: 5-Bromo-4-chloro-3-indolyl β-D-galactoside
Hap: Haplotype
LD: Linkage disequilibrium
