# Supplementary figures and images for: OsGATA16, a GATA Transcription Factor, Confers Cold Tolerance by Repressing OsWRKY45–1 at the Seedling Stage in Rice
Source: Rice (N Y). 2021 May 12;14:42. doi: 10.1186/s12284-021-00485-w (PMC8116401; doi:10.1186/s12284-021-00485-w)

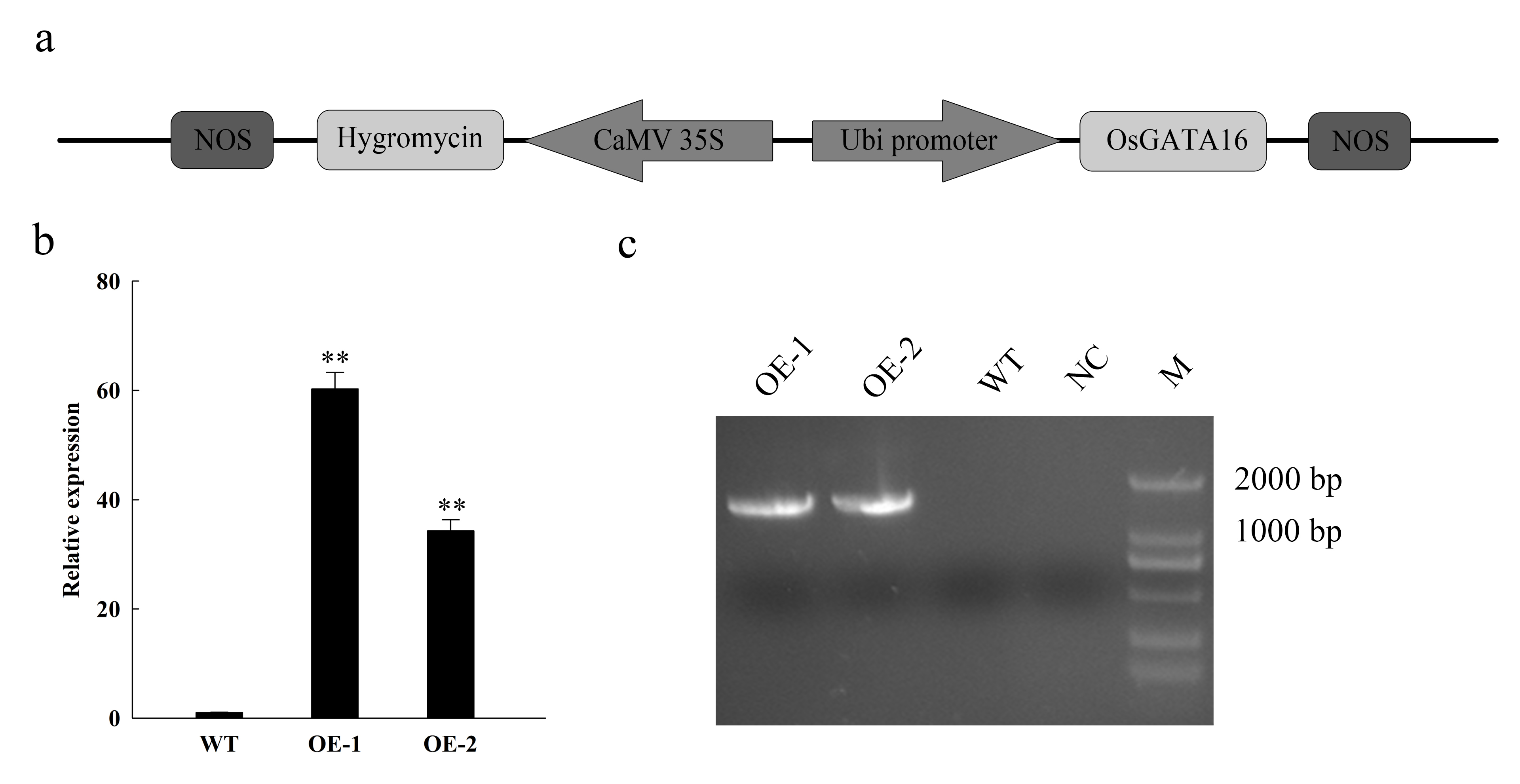

Supplement: Supplementary file 1 — Additional file 1: Fig. S1. Construction of OsGATA16 overexpression transgenic lines. (a) Schematic of recombinant overexpression plasmid with OsGATA16 under the control of a ubiquitin promoter. (b) Expression analysis of OsGATA16 in wild-type (WT) and overexpression (OE) lines by qRT-PCR. Data represent the mean ± SE from three replicates. Asterisks indicate significant differences in expression level (Student’s t-test, **p < 0.01). (c) Verification of transgenic OE lines by PCR. NC: negative control; M: 2000 bp marker. Fig. S2. Analysis of agronomic traits in wild-type (WT) and OsGATA16-overexpression (OE) lines under field conditions. (a) WT and OE plants at maturity. (b-c) Trait statistics for plant height (b) and hundred-grain weight (c) in WT and OE lines at maturity. Data represent the mean ± SE from three replicates. Fig. S3. Expression analysis of cold-sensitive genes in wild-type and OsGATA16-overexpression (OE) lines by qRT-PCR under normal and cold conditions. Data represent the mean ± SE from three replicates. [file 12284_2021_485_MOESM1_ESM.zip › Fig S1.jpg]

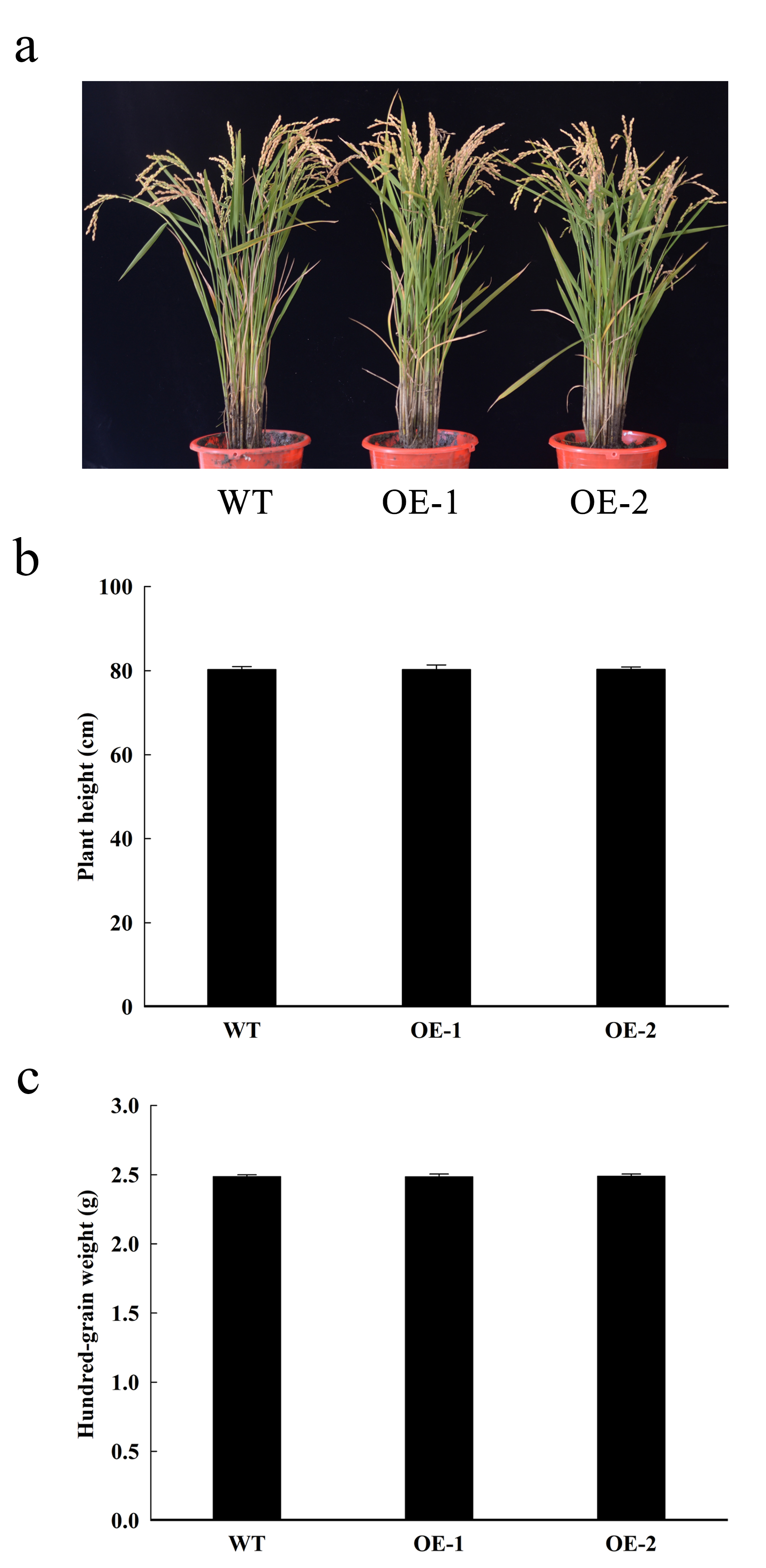

Supplement: Supplementary file 1 — Additional file 1: Fig. S1. Construction of OsGATA16 overexpression transgenic lines. (a) Schematic of recombinant overexpression plasmid with OsGATA16 under the control of a ubiquitin promoter. (b) Expression analysis of OsGATA16 in wild-type (WT) and overexpression (OE) lines by qRT-PCR. Data represent the mean ± SE from three replicates. Asterisks indicate significant differences in expression level (Student’s t-test, **p < 0.01). (c) Verification of transgenic OE lines by PCR. NC: negative control; M: 2000 bp marker. Fig. S2. Analysis of agronomic traits in wild-type (WT) and OsGATA16-overexpression (OE) lines under field conditions. (a) WT and OE plants at maturity. (b-c) Trait statistics for plant height (b) and hundred-grain weight (c) in WT and OE lines at maturity. Data represent the mean ± SE from three replicates. Fig. S3. Expression analysis of cold-sensitive genes in wild-type and OsGATA16-overexpression (OE) lines by qRT-PCR under normal and cold conditions. Data represent the mean ± SE from three replicates. [file 12284_2021_485_MOESM1_ESM.zip › Fig S2.jpg]

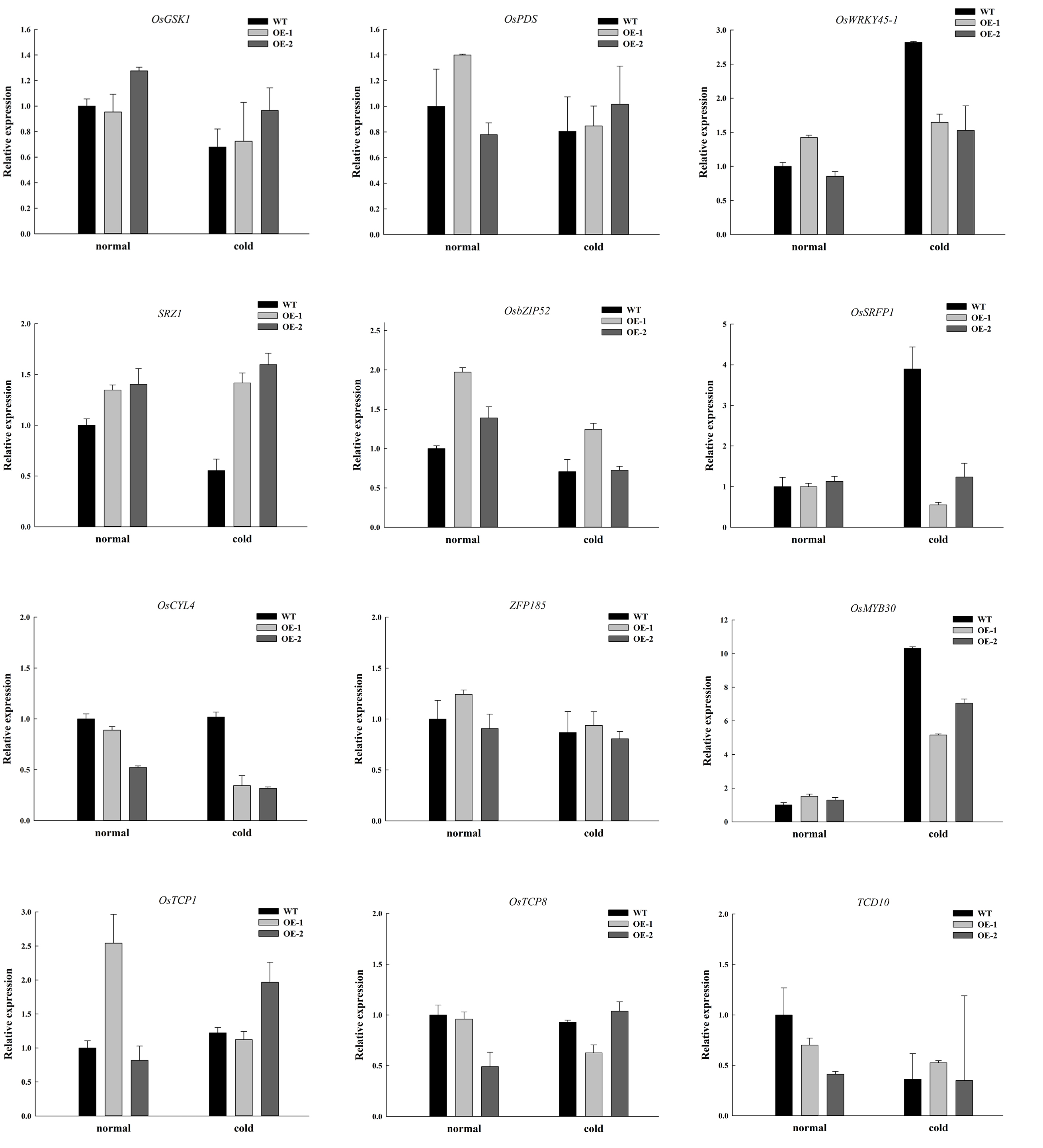

Supplement: Supplementary file 1 — Additional file 1: Fig. S1. Construction of OsGATA16 overexpression transgenic lines. (a) Schematic of recombinant overexpression plasmid with OsGATA16 under the control of a ubiquitin promoter. (b) Expression analysis of OsGATA16 in wild-type (WT) and overexpression (OE) lines by qRT-PCR. Data represent the mean ± SE from three replicates. Asterisks indicate significant differences in expression level (Student’s t-test, **p < 0.01). (c) Verification of transgenic OE lines by PCR. NC: negative control; M: 2000 bp marker. Fig. S2. Analysis of agronomic traits in wild-type (WT) and OsGATA16-overexpression (OE) lines under field conditions. (a) WT and OE plants at maturity. (b-c) Trait statistics for plant height (b) and hundred-grain weight (c) in WT and OE lines at maturity. Data represent the mean ± SE from three replicates. Fig. S3. Expression analysis of cold-sensitive genes in wild-type and OsGATA16-overexpression (OE) lines by qRT-PCR under normal and cold conditions. Data represent the mean ± SE from three replicates. [file 12284_2021_485_MOESM1_ESM.zip › Fig S3.jpg]
